# Gelatinous plankton is important in the diet of European eel (*Anguilla anguilla*) larvae in the Sargasso Sea

**DOI:** 10.1038/s41598-018-24388-x

**Published:** 2018-04-18

**Authors:** Daniel J. Ayala, Peter Munk, Regitze B. C. Lundgreen, Sachia J. Traving, Cornelia Jaspers, Tue S. Jørgensen, Lars H. Hansen, Lasse Riemann

**Affiliations:** 1 National Institute of Aquatic Resources, Technical University of Denmark, Kemitorvet, Kgs, Lyngby, Denmark; 2 Marine Biological Section, Department of Biology, University of Copenhagen, Helsingør, Denmark; 3 Evolutionary Ecology of Marine Fishes, GEOMAR - Helmholtz Centre for Ocean Research, Kiel, Germany; 4 Department of Environmental Science, Aarhus University, Roskilde, Denmark; 5 Department of Science and Environment, Roskilde University, Roskilde, Denmark

## Abstract

Limited insight into eel larvae feeding and diet prevents a holistic overview of the life-cycle of catadromous eels and an understanding of the ecological position of their early stages in marine waters. The present study evaluated the diet of larval European eel, *Anguilla anguilla* - a critically endangered species. Next-generation 18S rRNA gene sequencing data of Sargasso Sea eel larvae gut contents and marine snow aggregates was compared with a reference plankton database to assess the trophic relations of eel larvae. Gut contents of *A*. *anguilla* larvae were not well explained by the eukaryotic composition of marine snow aggregates; gut contents being dominated by gene sequences of Hydrozoa taxa (phylum Cnidaria), while snow aggregates were dominated by Crustacea taxa. Pronounced differences between gut contents and marine snow aggregates were also seen in the prokaryotic 16S rRNA gene composition. The findings, in concert with significant abundances of Hydrozoa in the study area, suggest that Hydrozoa plankton are important in the diet of *A*. *anguilla* larvae, and that consideration of these organisms would further our understanding of *A*. *anguilla* feeding strategies in the oligotrophic Sargasso Sea, which may be important for potential future rearing of *A*. *anguilla* larvae in captivity.

## Introduction

European eels, *Anguilla anguilla*, are long-lived migratory fish, which historically have been of great economic importance on the European continent^1^, and for over a century their biology and ecology has been the focus of numerous studies^2^. In recent decades, however, the population has undergone one of the fastest declines of fish populations ever reported^3^, and the species is currently listed as critically endangered by the International Union for Conservation of Nature^4^. Larval European eels, known as leptocephali, differ biologically from most other fish larvae. They have a large, transparent, and laterally-compressed body form, and a protracted larval duration, possibly lasting for 17–28 months^5^. Over the course of the extended larval period, the larvae drift over 5,000 km from the spawning area in proximity to frontal zones in the oligotrophic waters of the Sargasso Sea^2,6^ to the European and North African coasts^7^. Here they metamorphose and find habitats in estuarine or freshwater environments where they grow to adulthood. Adult eels subsequently undertake one-way migrations back to the Sargasso Sea^8–10^ to spawn and complete the cycle.

The functional role of eel larvae in the plankton of the Sargasso Sea is still largely unknown, despite a number of studies on their biology and ecology^6,11,12^. Studies of feeding based on field collected larvae present a number of problems^11,13^, see below, and studies on artificially reared ones is hampered by difficulties in raising them above the yolk-sac stage^14,15^. An understanding of the dietary preferences would contribute significantly to our understanding of their biology and aid aquaculture efforts to sustainably rear this species.

Several explorative approaches have aimed to determine the natural diet of anguilliform leptocephali. Guts of leptocephali usually appear empty, or contain a largely undistinguishable “mush” with little to no visual clues as to content. This was the reason for initial speculation that leptocephali subsisted on dissolved organic matter absorbed over their body surfaces^16,17^. Visual investigations later documented zooplankton fecal pellets and appendicularian houses in guts of some leptocephali species^18,19^, and early-stage Japanese eel, *Anguilla japonica*, leptocephali raised in aquaculture can feed on rotifers^20^. Fatty acid and lipid^21,22^ as well as isotopic examinations of eel larvae^13,22–24^ indicate that they feed on particulate organic matter (POM), which is dominated by organisms from lower trophic levels. Genetic studies of the DNA contained within stomach contents suggest, however, that small, gelatinous organisms could be an important part of the diet^25^, and a primary consumption of soft food particles/organisms is supported by morphological examinations of mouthparts and jaw-structures in conjunction with biomechanical modeling^26^. Hence, even today the diet composition of leptocephali remains unclear and it is unknown whether and to what extent they feed selectively on POM-aggregates and/or individual gelatinous organisms. The present study aims to shed light on this.

Gut-content investigations employing next-generation sequencing to evaluate stomach contents of predators have increasingly been employed to resolve trophic relationships and determine dietary breadth of organisms where traditional visual methods have proven difficult or impossible^27–29^. When experimental design, laboratory procedures, and bioinformatics are carefully carried out^30,31^, genetic sequencing can offer insights on dietary composition that cannot be achieved by morphological examination. Using this technique we investigated the diet of *A*. *anguilla* larvae sampled at the spawning area in the Sargasso Sea. Our goal was to link presence of specific gut contents with what was available in the immediate marine environment and examine if the composition of large particulate organic matter (POM visible by the naked eye; herein called *marine snow aggregates*) in the area was mirrored in the diet, suggesting that this was the primary food of larvae. To this end, we conducted extensive 16S and 18S rRNA gene sequencing of larval European eel gut contents and marine snow aggregates captured concurrently with, but discretely from, the sampling of eel larvae. Specimens of predominant planktonic taxa were also collected, and morphologically and genetically identified to create a site specific reference database covering some of the known gaps in public databases^25^. This study thus provides new insight into *A*. *anguilla* larval feeding by a genetic comparative examination of eel larvae dietary components in relation to various types of potential food items naturally occurring in the area where the early larvae are distributed.

## Results

**Figure 1:**
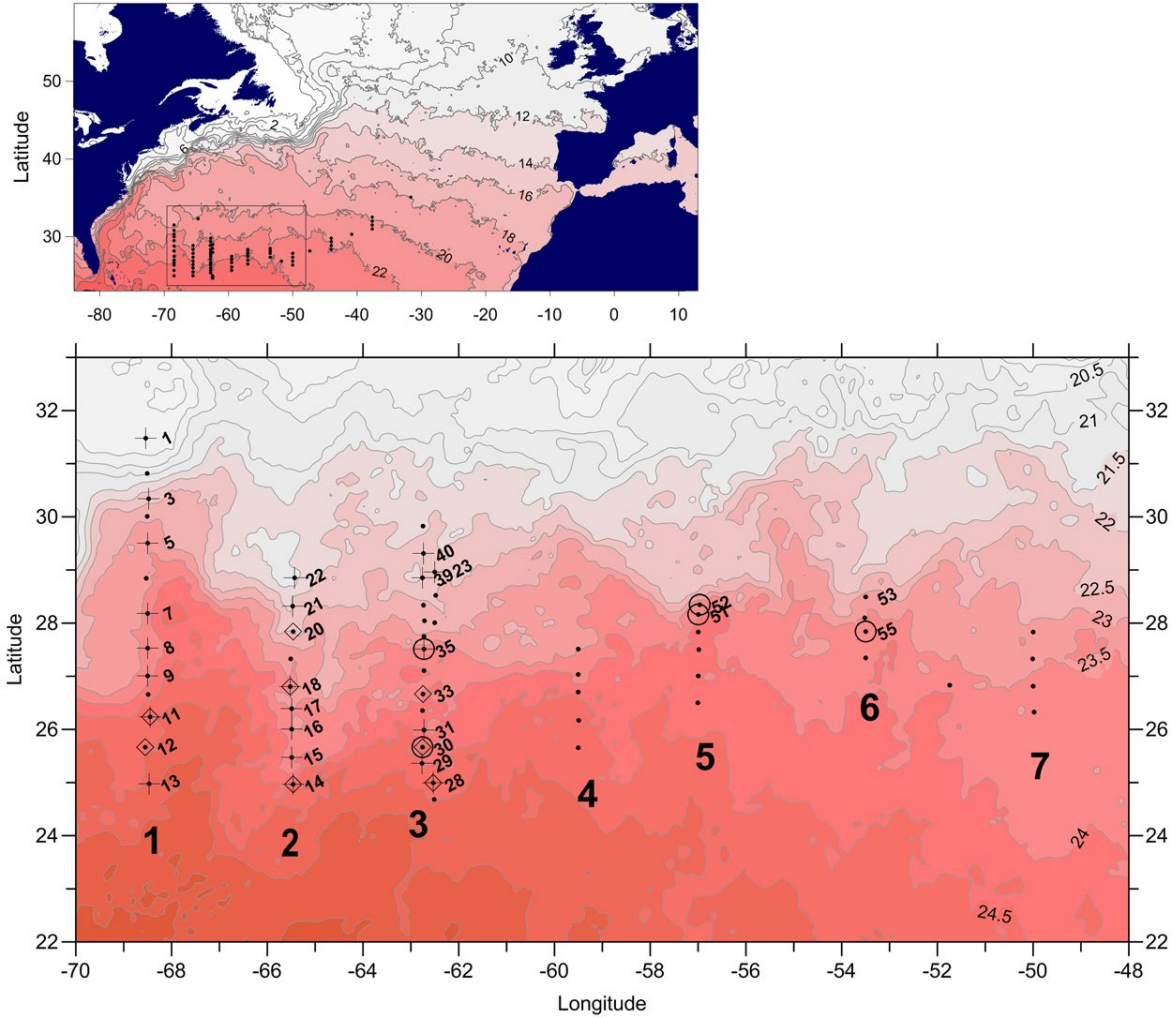
Satellite overview map of sampled stations across the northern Atlantic Ocean showing conditions on April 1, 2014. The map was made using Surfer® version 13 where the colored contour intervals denote sea surface temperature by 0.5 °C intervals. Black dots denote all sampled stations. Circles denote leptocephali stations, crosses denote plankton sampling stations, and diamonds denote marine snow stations.

During a cruise to the southern Sargasso Sea between March 30 and April 13, 2014, 82 stations were visited across a 500 × 2000 km wide region (Fig. 1). Leptocephali of *A*. *anguilla* were found at seven transects of sampling, and at a number of these stations larvae, plankton and marine snow particles were sampled (Fig. 1).

### Abundance and sizes of leptocephali and Hydrozoa

*A*. *anguilla* leptocephali used in the study were of a mean size of 15.2 ± 2.6 mm (range: 9.2 to 24.7; Supplementary Fig. S1), corresponding to an average age of 30 days^32^. The overall abundance of larvae varied substantially between stations, averaging 30 ± 44 × 10^−3^ m^−2^ (Supplementary Fig. S2). Hydrozoa biomass ranged between 3.8 to 30.4 mg C m^−2^ for the upper 200 m of the water column with an average carbon content of 16 ± 9.6 mg C m^−2^ (Supplementary Fig. S2). The occurrence of European eel (*A*. *anguilla*) was neither correlated with total Hydrozoa biomass (linear model, R^2^ = 0.04, p = 0.52, *n* = 13, Supplementary Fig. S2), nor with the major Hydrozoa biomass contributors, Hydromedusae (linear model, R^2^ = 0.01, p = 0.75, *n* = 13) or Siphonophorae (linear model, R^2^ = 0.04, p = 0.51, n = 13; Supplementary Fig. S3).

### Sequencing of 18S rRNA genes

The 18S rRNA gene sequences from guts of 75 European eel larvae and 31 marine snow aggregates passed the quality controls. They encompassed 64,352 and 411,444 reads, respectively, representing 225 OTUs from the eel guts and 769 OTUs from marine snow aggregates. Our custom-made reference database of 18 S rRNA genes from 75 individually picked zooplankton representatives and databases were used to assign taxonomy to OTUs.

### Composition of eukaryotes in eel larvae guts

**Figure 2:**
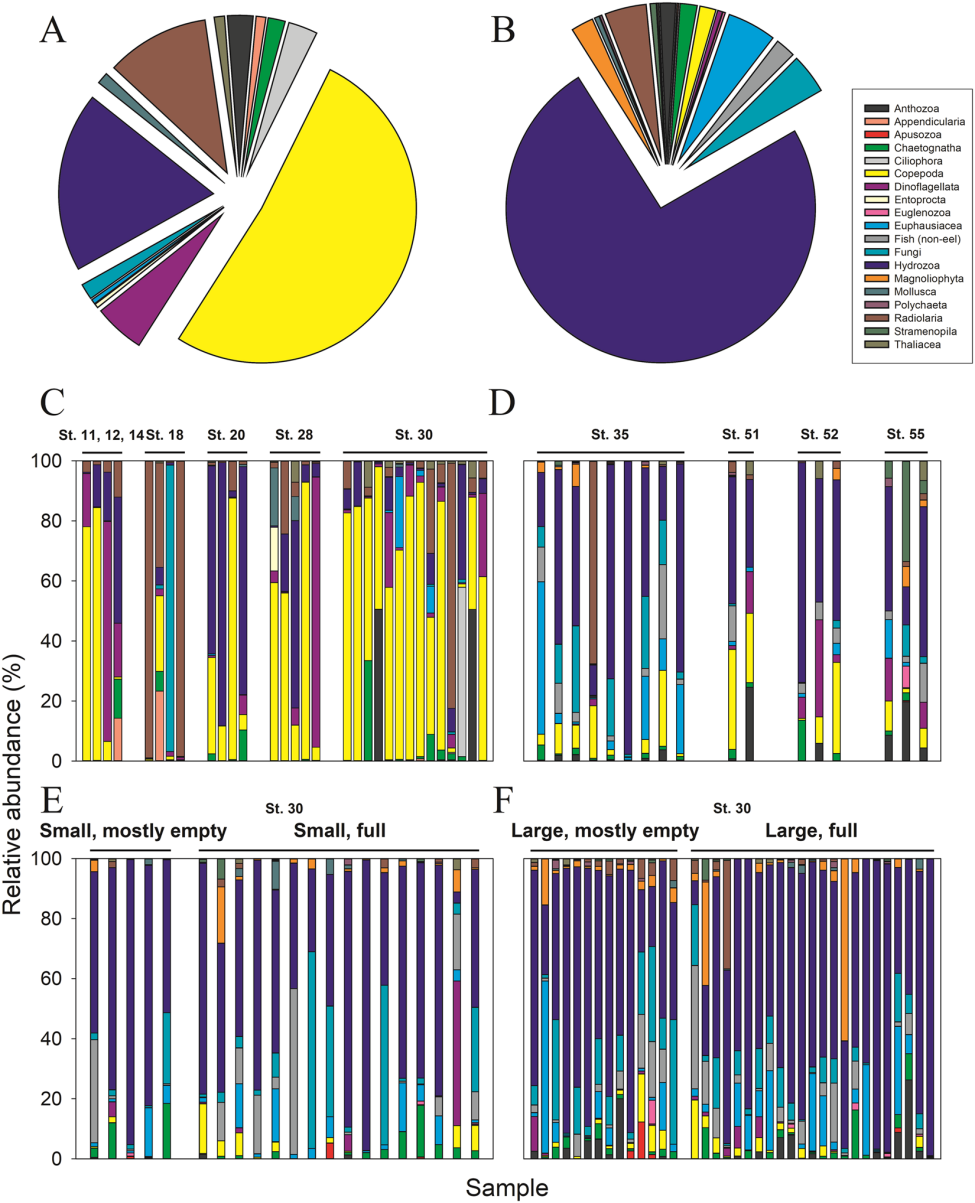
Composition of the eukaryotic taxonomic groups in marine snow aggregates and European eel leptocephali guts. (**A**) Summed overview of the marine snow aggregates (*n* = 31). (**B**) Summed overview of *Anguilla anguilla* gut contents (*n* = 75). (**C**) Composition of each marine snow particle separated by sampling station. (**D**–**F**) Composition of each leptocephali gut examined separated by sampling station; each column relates to an individual *A*. *anguilla* larvae. Station 30 additionally subdivided by larval size classes: “Small” < 15.2 mm, and “Large” ≥ 15.3 mm, as well as arbitrary assessments of gut content fullness. Plot based on top 50 most abundant OTUs accounting for 82% and 92% of all reads for marine snow and gut samples, respectively.

**Figure 3:**
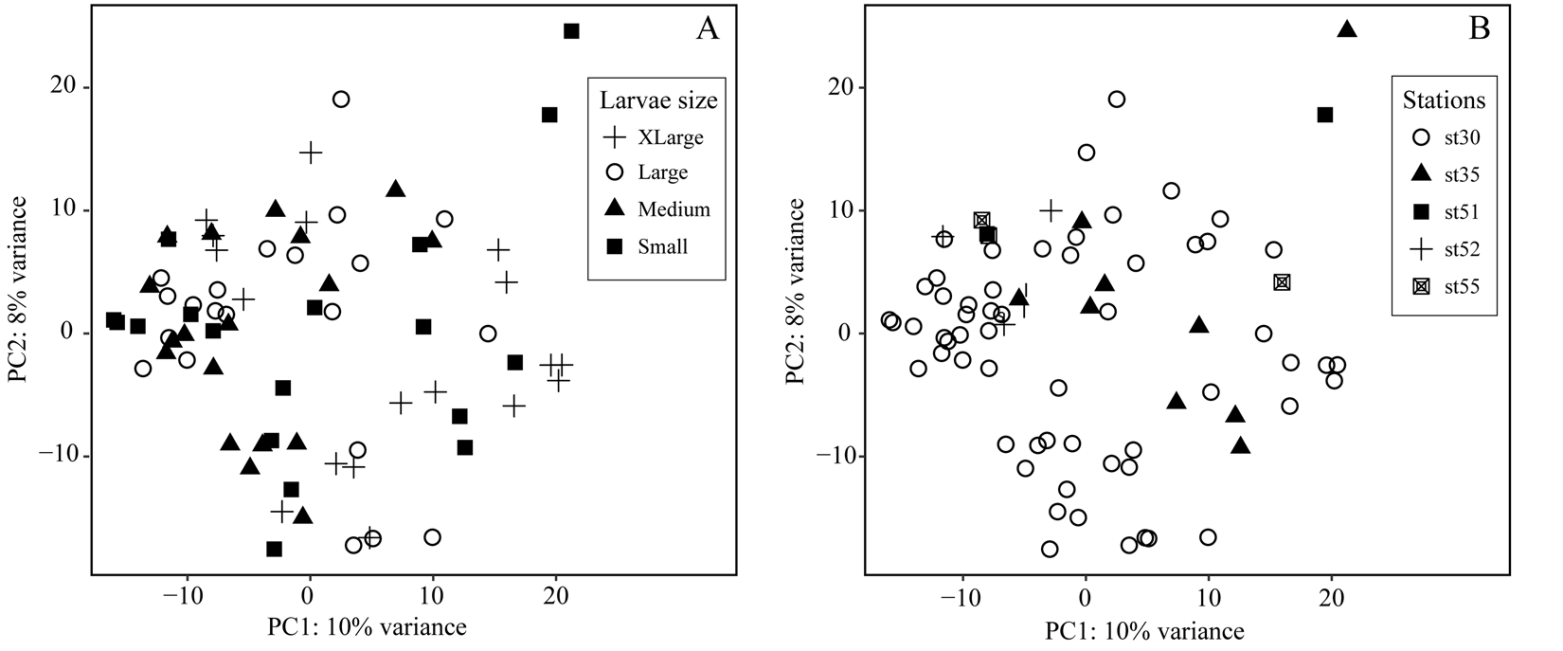
Principal component analysis of the 18S rRNA gene amplicon relatedness of *Anguilla anguilla* leptocephali gut contents (*n* = 75), partitioned according to four categories of leptocephali size (**A**), small (9.0–12.9 mm), medium (13.0–14.9 mm), large (15.0–16.9 mm), and Xlarge (17.0–24.7), and partitioned according to station (**B**).

Leptocephali gut OTUs were derived from 16 eukaryotic taxonomic lineages, of which the primary component was the Phylum Cnidaria. These gelatinous plankton organisms accounted for 76% of the reads and were composed of 98% Hydrozoa and 2% Anthozoa (Fig. 2B). At least 75% of the Hydrozoa sequences could be assigned to the group Siphonophora. Crustacea (copepods and euphausids) was the second most common group with an order of magnitude lower average abundance (7%). Less frequently occurring prey sequences included fungi (4%), radiolarians (4%), non-anguillid fish (2%), magnoliophytes (2%), chaetognaths (2%), stramenopiles (1%), dinoflagellates (1%), mollusca (1%), and a few other even less abundant groups. Genes of higher plants (e.g. magnoliophytes) are common in gene libraries from guts of plankton organisms^33,34^; however, at present we, and others^35^, suspect that they are potential contaminants and doubt that they originate from prey items. Pronounced variation was observed in composition between eel guts (Fig. 2D–F), but they were generally dominated by Cnidaria, and this group was encountered in every gut sample. Visual assessments indicated that 68% of eel larvae guts were full. None of the examined guts were completely empty, and none of them had visually distinguishable prey items when examined under the dissecting microscope. No systematic difference in composition was observed between full or mostly empty guts (Fig. 2E,F). Likewise, there were no systematic differences in gut content compositions between leptocephali size groups or between stations as suggested by a lack of distinctly separated groups in the PCA (Fig. 3).

### Composition of eukaryotes in marine snow aggregates

Marine snow aggregates were composed of eukaryotic OTUs from 13 taxonomic lineages, of which 52% of the reads on average per sample belonged to Crustacea (Fig. 2A). Cnidaria were the second-most abundant contributor (21% of reads), and this group was composed of 88% Hydrozoa and 12% Anthozoa reads. Of declining compositional contribution, with abundances from 11% to 0.4% of the reads, were radiolarians, dinoflagellates, ciliates, chaetognaths, fungi, molluscs, thaliaceans, appendicularians, and entroprocts (Fig. 2A). Individual marine snow aggregates exhibited substantial variation in their taxonomic compositions (Fig. 2C). For example, about half of the samples contained ≥50% Crustacea reads, while about a third only contained ≤10%. No systematic spatial differences in marine snow composition were discernible between stations as evidenced by the large degree of overlapping taxonomic groups by station (Fig. 2C), as well as the lack of distinctly separated stations in a PCA (data not shown).

### Compositional similarity between eel gut contents and marine snow

**Figure 4:**
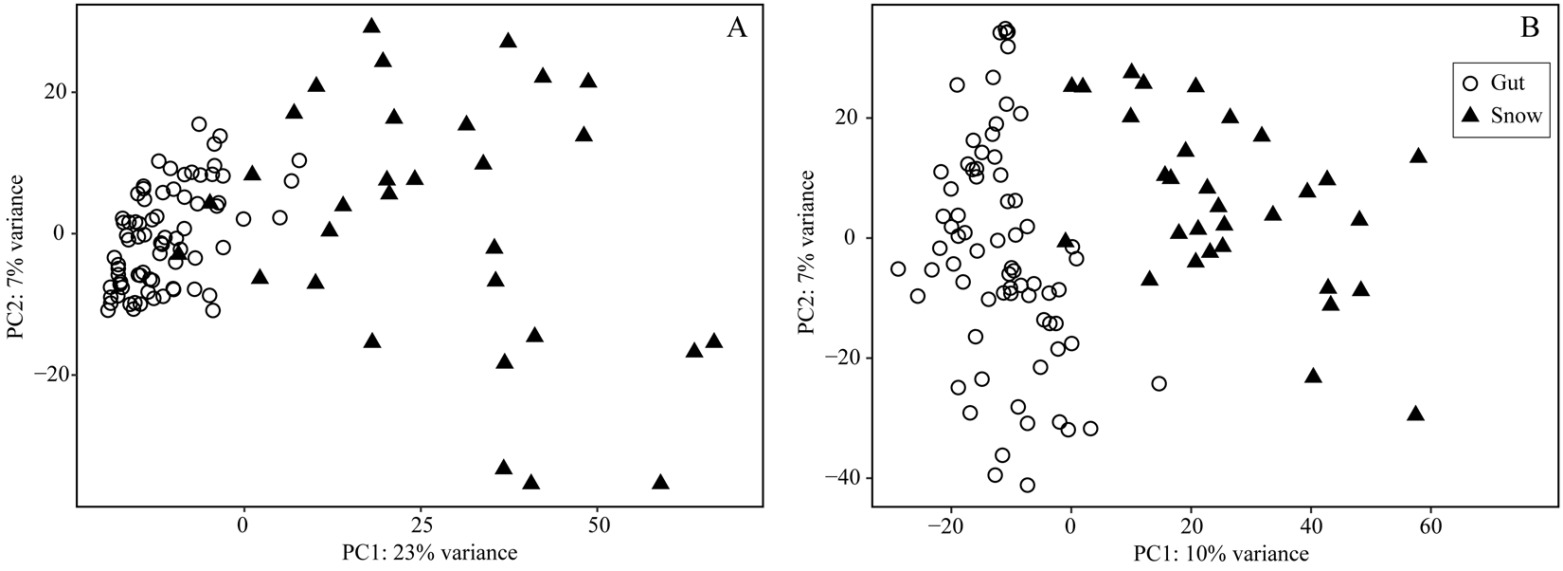
Principal component analyses of *A*. *anguilla* gut contents and marine snow particle compositions: (**A**) Eukaryotic (18S rRNA genes) and (**B**) Prokaryotic (16S rRNA genes) composition. Triangles indicate individual leptocephali gut contents and circles indicate individual marine snow aggregates.

The relative contribution of predominant OTUs as well as the overall composition differed between eel guts and marine snow aggregates. This was the case when comparing all samples (Fig. 2), and when comparing eel guts and marine snow aggregates obtained from the same station (station 30; Supplementary Fig. S4). In particular, the major and shared taxonomic groups showed clear differences in relative contribution, namely Cnidaria and Crustacea that accounted for 76% and 7% in eel guts but 21% and 52% in the marine snow samples (Fig. 2A,B). When comparing overall eukaryotic composition in the samples by PCA, only a marginal overlap was observed between eel larvae gut contents and marine snow aggregates (Fig. 4A), and the GLM analysis showed a significant difference between eel gut content and marine snow particles (p ≪ 0.0001). To further investigate this difference, a GLM analysis was applied to identify the OTUs, which contributed significantly to the differences between gut and marine snow samples. Of the OTUs contributing to the difference between gut and snow samples, the majority were related to Crustacea (Supplementary Table S1).

After quality control, the 16S rRNA gene sequences from guts and marine snow aggregates encompassed 179,466 and 88,203 reads, respectively. There were large differences in prokaryotic composition between leptocephali gut contents and marine snow aggregates when looking at the phylum and sub-phylum level (Supplementary Fig. S5). For instance, cyanobacteria (*Trichodesmium*, *Prochlorococcus*, or *Synechococcus*) accounted for >0.5% of the reads in 90% of the marine snow samples, but only in 25% of the eel guts (Table 1). Also, Alphaproteobacteria accounted for >20% of the reads in 39% of the marine snow samples, but only showed a similar dominance in 13% of the eel guts. In particular, the Rhodobacterales order was predominant, but variable, on marine snow aggregates. Overall, the prokaryotic composition differed between the eel larvae guts and marine snow, as indicated by generally disparate groups in the PCA (Fig. 4B).

**Table 1:** Relative abundances, as proportion of 16S rRNA gene reads, of selected prokaryotic taxa in eel guts and marine snow aggregates. *Indicates proportion (%) of samples where Cyanobacteria account for >0.5% of reads. **Indicates proportion (%) of samples where Alphaproteobacteria account for >20% of reads.

	*n*	Phylum Cyanobacteria	Genus *Trichodesmium* sp.	Genus *Synechococcus*	Genus *Prochlorococcus*	Samples with Cyanobacteria*	Order *Rhodobacterales*	Samples with Alphaproteo-bacteria**
Eel guts	71	1.2 ± 4.2	0	0.4 ± 2.7	0.0 ± 0.4	25	1.3 ± 3.1	13
Marine snow aggregates	31	23 ± 30	13 ± 26	1.8 ± 8.4	2.0 ± 6.8	90	11 ± 17	39

## Discussion

The guts of European eel larvae, as analyzed by 18S rRNA gene sequencing, were on average dominated by Hydrozoa, gelatinous zooplankton of the Phylum Cnidaria, which accounted for 76% of all reads. Remaining reads were affiliated with an assortment of other marine plankton taxa. Marine snow aggregates had a broad composition of plankton taxa, wherein Crustacea accounted for 52% of the reads, followed by 21% contribution by Cnidaria, of which 88% was from Hydrozoa. Such diverse makeup is consistent with the diverse components thought to constitute marine snow^36–38^. Both *A*. *anguilla* gut contents and marine snow aggregates had contributions from Cnidaria and Crustacea taxa but their relative contributions to the two sample types were diametrically opposed. We speculated whether this difference could be due to bias, where our marine snow sampling could have included copepod taxa known to thrive on the surface of marine snow (e.g. species of Oncaea, Microsetella and Oithona^39^) whereas these could have escaped when the marine snow particle was preyed upon by eel larvae. However, the highest proportion of total reads (14%) among the Crustacea taxa belonged to Clausocalanus species, which were also the predominant taxa in terms of biomass in the water column (Lundgreen *et al*., unpublished). In fact, Microsetella species were not among the top 50 OTUs of the eel guts. *Oithona* spp. accounted for 7% of the reads but it was also a major contributor to copepod biomass in the water column. Taken together, we do not find it likely that the marked difference in the proportion of Crustacea on marine snow (52% of reads) versus eel larvae guts (7% of reads) is caused by a sampling bias. While the picture is not complete, the 18S rRNA gene sequencing data suggests that marine snow is not likely to constitute the main part of the leptocephali diet, as marine snow consumption cannot explain the high percentages of gelatinous plankton sequences in the gut contents. This conclusion is supported by the 16S rRNA gene analysis, which showed distinct prokaryotic compositions of marine snow aggregates and eel guts (Fig. 4B). The potential presence of a permanent microbiota in eel guts could have contributed to this observed difference in 16S rRNA gene composition between marine snow and gut samples; however, we find this option unlikely since (1) both eukaryotes and prokaryotes, respectively, showed a similar distinct composition of marine snow aggregates and eel guts (Fig. 4A vs. B), and (2) this cannot explain the major differences observed for specific groups (Table 1). Hence, also the 16S rRNA gene analyses indicate that marine snow aggregates are not a predominant food source for eel larvae.

Our findings of a potentially Hydrozoan-dominated diet are consistent with earlier studies suggesting that gelatinous zooplankton^26,40^, and Hydrozoa in particular^25^, could be of importance in the diet of young *A*. *anguilla* larvae in the Sargasso Sea. The prevalence of Hydrozoa was highly variable between eel guts, accounting for 4–99% of the reads per leptocephali gut. Since gelatinous plankton is relatively rapidly digested in fish guts^41^ this could have contributed to the variation observed between examined guts, along with supposedly variable digestion rates between different plankton taxa. Also, although 18S rRNA gene metabarcoding as done here may be considered semi-quantitative^42^, differences in gene copy number per genome and in genome sizes between prey items, or preferential amplification of some DNA templates^43^, could have biased the estimate of Hydrozoa in the guts. Moreover, the extent to which degradation of gut contents took place during the 15–60 min on ice prior to ethanol fixation is unknown. On the other hand, the fast degradation rates for gelatinous taxa in fish guts^41^ combined with their low DNA content relative to bio volume, if assuming that the extremely low carbon content of gelatinous taxa^44,45^ is mirrored in a low DNA content, may have caused an underestimation of the contribution of Hydrozoa to the eel diet. Hence, the importance of Hydrozoa in the diet of the examined European eel larvae is based on conservative assumptions, however, their actual importance for growth of early life stages need further investigations via controlled growth-feeding experiments. Unfortunately, at the current stage of knowledge regarding survival and feeding of European Eel larvae, such experiments are not feasible.

To investigate if the observed densities and biomasses of calycophoran siphonophores (accounting for 75% of the Hydrozoa sequence reads in guts) are high enough to account for the energetic demand of eel larvae in the investigated area, we assume that a 15 mm sized larvae would be able to tear apart the siphonophores (average size 5 mm) and eat the pieces. With relatively large-sized prey as siphonophores, compared to e.g. copepods in the area, eel larvae would have a relatively long distance of reaction and hence be able to search a large volume for this type of prey. We assume that the reaction distance could be >35 mm (distances seen for herring larvae preying on large sized copepods^46^), and that the search will be within a hemisphere above the head^46^. Setting the swimming speed to 1 body length s^−1^ we estimate the potential searched volume of larvae for siphonophores to >1.3 m^3^ during a 12 h feeding period. Siphonophores of the sub-order calycophorae were observed at maximum densities of 4 ind. m^−3^ and their carbon content estimated to 0.4 mg C m^−3^ (based on size^47^) or 2 mg C m^−3^ (based on carbon measurements; Jaspers *et al*., unpublished). Eel larvae would then, according to the calculations, be able to consume about 5 siphonophores during a feeding day. The minimum energetic requirement for leptocephali of our average size and weight is estimated to about 8.4 J ind.^−1^ day^−1^ (15 mm and 0.02 g wet mass, respectively; J day^−1^ = 72.64 wet mass^(0.55)^)^48^. As gelatinous zooplankton carbon content is maximally 15% of dry weight^49^ and energy content per dry weight is 2.0 to 5.0 kJ g^−1^; ^50,51^, this leads to a potential contribution of siphonophores to the diet of eel larvae of 0.7 to 1.6 or 3 to 8 times the larval energy demand considering size or weight based carbon content estimates, respectively. Hence, from this calculation, siphonophores at the present sizes and densities appear able to meet the entire energy demands of leptocephali.

From our study, we cannot discriminate whether the Hydrozoa detected in *A*. *anguilla* guts were eaten opportunistically or whether they were selectively targeted prey. If leptocephali as previously suggested preferentially consume marine snow aggregates^13,21^, and get the Hydrozoa from these, then we would expect that the proportion of Hydrozoa in the guts should be in accordance with its proportion in marine snow aggregates. This is not the case, and further our direct measurement of molecular composition in snow particles showed no dominance of Hydrozoa. Genes from gelatinous zooplankton have been found in guts of lobster larvae^28,52^, which are also highly flattened and have a protracted larval life stage, like eel larvae. Moreover, visual and isotope analyses of guts show that many marine fish species occasionally or predominantly feed on jelly plankton^53–56^; hence, it is not unlikely that Hydrozoa are important in the eel larval diet.

Studies investigating isotope composition of leptocephali have shown relative low proportions of isotopes ^13^C versus ^12^C and ^13^N versus ^14^N, this taken as indication of primary feeding on POM, which could be dominated by organisms from lower trophic levels^13,21–24^. These findings seemingly contradict our suggestion of a large contribution of Hydrozoa in the diet since Hydrozoans are primarily preying on copepods, hence, are generally feeding at higher trophic levels, and are therefore expected to possess higher isotope proportions. However, there are indications that the measured low isotope values of leptocephali do not exclude a feeding on Hydrozoans. Firstly, oligotrophic areas, like the Sargasso Sea, with relatively large abundance of *Trichodesmium* and *Richelia* appear to generally have low ^15^N values due to high nitrogen fixation rates^57^. Secondly, in studies investigating isotope ratios of zooplankton communities, the Hydrozoa appear to have lower isotope ratios than other zooplankton organisms, e.g. copepods^24,53^. Hence, conclusions drawn in the present study, and those of recent isotope analyses (e.g. refs^13,23,24^), may not be mutually exclusive, especially when considering that analysis of size-fractionated particles (cf. refs^13,23,24^) is substantially different than our analysis of discretely sampled marine snow aggregates.

Our finding of high proportions of Hydrozoa DNA sequences in the eel larvae guts suggest selective feeding on Hydrozoa, a possibility supported by estimations that indicate sufficient densities of these organisms to accommodate *A*. *anguilla* feeding demands in the Sargasso Sea. Based on the distinctly different eukaryotic and prokaryotic compositions in eel guts and marine snow, we infer that although marine snow aggregates may contribute to the larval *A*. *anguilla* diet, these are not likely to be the main food source for *A*. *anguilla* larvae.

Finding adequate and abundant sources of nutrition is necessary for growth and survival during the larval stage which is believed to be a critical period within the life-cycle of fish^58^, setting the size of recruitment to the adult stock. The present dietary analysis provides novel information of great significance for our perception of larval feeding abilities at the spawning grounds in the Sargasso Sea, these determining the early survival of the critically endangered European eel. Additionally, the insights into feeding habits will be of value for the attempts of rearing this species in aquaculture, considering the current problems in sustaining larvae after first-feeding.

## Methods

### Sampling and identification procedures

Leptocephali were sampled with a 3.5-m-diameter ring net, equipped with a 25-m-long, 560 µm net, with a 300 µm meshed 1 m long cod-end (MIK net). Oblique trawls were carried out at a ship speed of 2.5 knots to the depth of 200 m, which is expected to cover the majority of the eel larval abundance^12^. Flowmeters were used to assess the filtered volume. Once on-board, the plankton sample was immediately placed on ice, eel larvae were sorted from the plankton, and potential European eel leptocephali preliminarily identified using myomere counts together with location of the last vertical blood vessel. Potential European eel larvae were measured to the nearest 0.1 mm, digitally photographed, rinsed twice in 0.2 µm filtered seawater, and stored individually in 96% ethanol for subsequent genetic species verification. Larvae were generally fixed in ethanol within 15–30 min of sampling; a maximum of 60 min occasionally transpired at stations with either many eel larvae, or which contained large amounts of Sargassum. Species-identity of *A*. *anguilla* larvae was confirmed genetically using analysis of the mitochondrial cytochrome *b* gene and microsatellite genotyping^25^. The gut from genetically-confirmed *A*. *anguilla* larvae from 5 stations (Fig. 1) was excised using a sterile disposable hypodermic needle (Microlance #3, Becton Dickinson, New Jersey, USA) in a sterile petri dish under a dissecting microscope to minimize contact with the larval skin surface, and transferred whole to a DNA-free tube containing 100 µl of 96% ethanol. Visual assessments of each individual eel gut’s relative fullness or emptiness were made during gut extraction. As no larvae exhibited a strictly empty gut, larvae were divided into categories of either “full” or “mostly empty” for comparison. Filled guts were encountered in both larvae that were sampled day and night. This could imply feeding that is not restricted to only occur during the daytime or nighttime if gut retention times are short. The larvae are visual feeders (large eyes), and hence irrespective of the time of sampling, we expect the stomach content to stem from feeding when larvae are predominantly in the 110–160 m layer. While this depth coincides with their observed peak daytime abundance (Munk *et al*., in revision) the more widespread larval distribution in the water column during nighttime could also include feeding at this depth range, despite the generally higher peak abundance in the uppermost depth layer during nighttime.

Marine snow aggregates were collected at 8 stations (Fig. 1) using a custom made 90 µm ‘‘Appinet” with a 1 m mouth diameter. The 1.5 m long net ends in a canvas bag holding a large plexi glass cod end (diameter: 30 cm, height: 46 cm) with a volume of 32.5 L. The construction was designed to minimize damage to fragile planktonic organisms and marine snow aggregates. Sampling was done with vertical tows from the sub-surface fluorescence max to the surface with a speed of 0.1 m s^−1^. After retrieval, the canvas bag holding the transparent cod end was zipped off, gently carried to the lab, and immediately analyzed for marine snow particles on a light table. Undisturbed marine snow aggregates were individually removed using a glass pipette with a suction tube, rinsed with 0.2 µm filtered seawater, photographed, and stored in Eppendorf tubes at −80 °C.

To match stomach content of eel larvae to the prey field, gelatinous and other zooplankton organisms from all major taxa, including the most abundant species in the depth layer where eel larvae were found, were identified and sampled for DNA sequencing. In total 75 plankton organisms sampled at 22 stations (Fig. 1) were used to generate a reference-plankton database. Depending on the fragility of the animals, samples for DNA extraction were attained by (i) Appinet, (ii) a slowly horizontally towed, 335 µm multiple opening and closing Multinet “midi” (HYDROBIOS, Kiel, Germany), or (iii) a MIK net. Filtered water volumes ranged from 50 to 300 m^3^ for net (i) and (ii), to >10,000 m^3^ for net (iii). For potential prey of the eel larvae, the euphotic zone was sampled using net (i) integrating the upper water column above the sub-surface fluorescence max, net (ii) sampling discrete depth strata from 0–50 m, 50–100 m and 100–200 m, while net (iii) obliquely sampled the entire upper 200 m of the water column. Hydrozoa biomass was estimated from depth stratified abundance data obtained by net ii) and published length to carbon regressions^47^. All samples were analyzed alive for hydromedusae and larger gelatinous zooplankton taxa (Jaspers *et al*. in preparation), while abundances of siphonophores were based on analyses of preserved samples (Li *et al*. submitted). All handling of animals was carried out in accordance with relevant guidelines and regulations, as approved by the University of Copenhagen.

### Genetic analysis of eel gut contents, marine snow aggregates, and plankton specimens

The 16S and 18S rRNA marker genes were examined with Illumina MiSeq to discern prokaryotic and eukaryotic compositions, respectively, of eel guts and marine snow aggregates, while Sanger sequencing of 18S rRNA genes from zooplankton specimens was used to generate the reference database. Total genomic DNA was extracted from each eel gut using the E.Z.N.A. Tissue DNA Kit (OMEGA Bio-Tek, Georgia, USA), and from the marine snow aggregates using phenol-chloroform extraction^59^, and quantified (PicoGreen®, Quant-iT^TM^, Invitrogen, ThermoFisher Scientific, Massachusetts, USA). The V7 region of the 18 S rRNA genes was PCR amplified using 0.05 units µl^−1^ MyTaq DNA polymerase (Saveen & Verner AB, Limhamn, Sweden), ca. 0.08 ng µl^−1^ DNA template, and 0.5 µM of each of universal primers UnivF-1183mod and UnivR-1443mod^60^ indexed for Illumina sequencing in 25 µl reaction volumes. A specially designed blocking primer, modified with a C3-spacer to prevent elongation (*cf*.^61^), was introduced to each PCR mixture with an eel gut DNA template to block the amplification of *A*. *anguilla* DNA (5′-CATCACAGACCTGTTATTGCTCAATCTCGTGTGGCTGAACG3–3′; 3 = C3-spacer), while still allowing amplification of the full range of potential prey organisms identified in Riemann *et al*.^25^. Sequences from a range of potential prey organisms found by Riemann *et al*.^25^ were aligned and analyzed as done by Leray *et al*.^62^ to facilitate blocking primer design. The blocking primer was added at a ratio of 0.8:1 relative to the reverse primer, after trials to determine a sufficient host-blocking effect utilizing the least amount of blocking primer. Blocking primer trials were conducted against DNA of genetically-confirmed *A*. *anguilla* specimens from a previous study^63^. PCR conditions were 95 °C for 2 min followed by 30 cycles of 94 °C for 30 s, 57 °C for 30 s, and 72 °C for 45 s, and termination at 72 °C for 10 min. Triplicate PCR reactions per sample were pooled, purified (Agencourt AMPure XP magnetic bead system, Beckman Coulter Life Sciences, Indiana, USA), and quantified using PicoGreen. Finally, PCR amplicons from eel guts and marine snow aggregates were pooled at equimolar concentrations, and submitted for paired-end sequencing on an Illumina MiSeq V2 2 × 250 (University of Copenhagen, Denmark).

The V3 and V4 regions of the 16S rRNA genes were PCR amplified with the universal primers 341F and 806R^64^. Reactions were set up in 20 µl using 0.03 units µl^−1^ Phusion HotStart II polymerase and HF buffer (Thermo Fisher, Massachusetts, USA), ca. 0.1 ng µl^−1^ DNA template, and 100 ng µl^−1^ bovine serum albumin (Bioron, Ludwigshafen, Germany). PCR conditions were 98 °C for 30 s followed by 35 cycles of 98 °C for 10 s, 56 °C for 20 s, and 72 °C for 30 s, and final elongation at 72 °C for 5 min. Sequencing and indexing adaptors were added to the PCR products by a second round of PCR, performed by applying the Nextera indexing kit (Illumina Inc. San Diego, California, USA) under the conditions described earlier^65^, but modified to 5 μL of the 1st round PCR product as the template and 17 cycles. PCR products were purified with MagBio magnetic beads (MAGBIO GENOMICS, Maryland, USA). PicoGreen quantification was used for molarity estimation and samples were paired-end sequenced on an Illumina MiSeq platform using the MiSeqV2 500 cycles sequencing kit (Aarhus University, Roskilde, Denmark).

DNA for the reference database was extracted (E.Z.N.A. Tissue DNA Kit) from individual planktonic organisms, quantified (PicoGreen), and PCR amplified using universal 18 S rRNA primers F-1183mod and R-1631a according to Hadziavdic *et al*.^60^. To increase the reference-database coverage, DNA from 14 genetically-confirmed fish specimens from 13 families, including 3 common eel families, was amplified with the same primers. These fish represent the most abundant fish species known to inhabit this area of the Sargasso Sea at the time of sampling^66^. PCR amplicons were purified (E.Z.N.A. Cycle Pure Kit, OMEGA Bio-Tek, Georgia, USA) and Sanger sequenced commercially (Eurofins, Ebersberg, Germany).

### Bioinformatics and taxonomic assignments

After sequencing, 18S rRNA gene Illumina sequence reads were assembled and trimmed to their median length, before trimming, of 224 nucleotides, and de-multiplexed (quality score of Q30) using QIIME v1.9^67^. Removal of singletons and clustering of operational taxonomic units (OTUs) at 99% similarity was done in USEARCH v8.1.1756^68^ using the UPARSE-OTU algorithm^69^ with implicit chimera check. Taxonomy was assigned using the SILVA v. 119 database^70^ and BLASTN^71^. BLASTN criteria was a coverage score >98%, with BLASTN homology >95%, and 100% identity similarity over ≥100 bp. The taxonomy of the highest ranking match was then assigned to the OTU. In instances where the highest match included several organisms or species with equal similarities over the same number of bp, taxonomy was assigned using the lowest common taxonomic denominator for the grouping. Remaining ambiguous or unidentified/unknown OTUs were aligned to our custom plankton-database, first by aligning all sequences in CLUSTAL W, then via placement in a phylogenetic tree constructed in MEGA6^72^. Identification of Actinopterygii (fish) OTUs was similarly done in this manner. OTUs only occurring once in the total dataset or which included <9 reads in total, were excluded, as were *A*. *anguilla* sequences, and any Actinopterygii OTUs with ≤2% (maximally 4 bp difference) dissimilarity to *A*. *anguilla*.

16S rRNA gene sequence reads were split into samples by unique custom barcodes. All subsequent steps were performed in CLC genomics workbench 9.5.3 using the microbial genomics plugin. A maximum of 10,000 reads was used per sample. Reads were merged and trimmed for adaptors, low quality and short reads. Samples with fewer than 1000 reads were not further processed and all reads were trimmed to the same length. Merged reads were clustered to OTUs on a 97% identity level to the ARB-SILVA v119 database, removing chimeras in the process. Singletons were removed before further analysis.

### Statistical analysis

Statistical analyses were carried out in R^73^. To address a negative binomial data structure in the 18S and 16S rRNA amplicon data, OTU abundances were normalized using DESeq2 1.14.1^74^, and community compositions were analyzed using principal component analysis (PCA). Compositional differences between eel gut contents and marine snow aggregates were tested using generalized linear models (GLMs) and mvabund 3.12^75^. Furthermore, an ANOVA was applied to the GLM models to identify the OTUs, which contributed significantly to the differences between gut and marine snow aggregates. All p-values reported are adjusted using Holm’s stepdown multiple testing controlling for the family wise error rate, as implemented in the mvabund package.

### Data availability

Raw nucleotide sequences for the 18S and 16S rRNA gene analyses have been archived in the Short Read Archive, Genbank, National Center for Biotechnology Information or at the European Nucleotide Archive at EMBL-EBI under accession numbers SRR6157677 and PRJEB20115, respectively. Nucleotide sequences for the reference database have been deposited in GenBank under accession numbers KY594837-KY594911.

## Electronic supplementary material

Supplementary Information
