# Supplementary material for: Gelatinous plankton is important in the diet of European eel (Anguilla anguilla) larvae in the Sargasso Sea
Source: Sci Rep. 2018 Apr 18;8:6156. doi: 10.1038/s41598-018-24388-x (PMC5906606; doi:10.1038/s41598-018-24388-x)
Supplement: Supplementary file 1 — Supplementary Information [file 41598_2018_24388_MOESM1_ESM.docx]

**Electronic supplementary material**

**“Gelatinous plankton is important in the diet of European eel (*Anguilla anguilla*) larvae in the Sargasso Sea”** by Daniel J. Ayala, Peter Munk, Regitze B. C. Lundgreen, Sachia J. Traving, Cornelia Jaspers, Tue S. Jørgensen, Lars H. Hansen and Lasse Riemann

Table S1. List of the top operational taxonomic units (OTUs, clustered at 99% similarity) that contribute significantly to composition differences between *Anguilla anguilla* gut and marine snow samples. The OTUs are listed according to the adjusted p-values from the generalized linear model (GLM) analyses. *Nearest relative in GenBank. If no nearest relative is noted, taxonomy was determined from phylogenetic trees (Lundgreen et al., unpublished).

| #OTU | Test size | p adjusted | Taxonomy | Nearest relative * | Similarity, % | Coverage, % |
| --- | --- | --- | --- | --- | --- | --- |
| OTU_1805 | 85.38748 | 1.00E-04 | Copepoda | *Clausocalanus furcatus* | 98 | 100 |
| OTU_5 | 76.4959 | 1.00E-04 | Copepoda | *Paracalanus parvus* | 99 | 100 |
| OTU_7 | 101.6175 | 1.00E-04 | Copepoda | *Haloptilus longicornis* | 99 | 100 |
| OTU_13 | 80.10632 | 1.00E-04 | Radiolaria | *Xiphacantha alata* | 99 | 100 |
| OTU_14 | 79.9251 | 1.00E-04 | Copepoda | - | - | - |
| OTU_32 | 78.26328 | 1.00E-04 | Dinoflagellata | *Gymnoxanthella radiolariae* | 100 | 100 |
| OTU_21 | 74.55008 | 1.00E-04 | Copepoda | *Lucicutia ovaliformis* | 99 | 100 |
| OTU_8 | 72.80797 | 2.00E-04 | Copepoda | *Calanus sinicus* | 100 | 100 |
| OTU_6 | 69.13703 | 2.00E-04 | Copepoda | *Oithona similis* | 99 | 100 |
| OTU_10 | 70.58102 | 2.00E-04 | Copepoda | *Oncaea* sp. | 98 | 100 |
| OTU_1255 | 66.41127 | 3.00E-04 | Copepoda | *Oithonidae* sp. | 99 | 100 |
| OTU_526 | 63.98246 | 3.00E-04 | Radiolaria | *Acanthometron* sp. | 99 | 100 |
| OTU_1240 | 66.95246 | 3.00E-04 | Copepoda | *Clausocalanus furcatus* | 99 | 99 |
| OTU_4 | 63.05308 | 4.00E-04 | Hydrozoa | *Liriope tetraphylla* | 100 | 100 |
| OTU_31 | 62.31555 | 4.00E-04 | Dinoflagellata | Uncultured symbiont | 100 | 100 |
| OTU_24 | 61.17682 | 4.00E-04 | Thaliacea | *Dolioletta gegenbauri* | 100 | 100 |
| OTU_99 | 62.61122 | 4.00E-04 | Copepoda | *Paracyclopina nana* | 98 | 100 |
| OTU_41 | 53.80944 | 0.0014 | Radiolaria | *Dorataspis loricata* | 100 | 100 |
| OTU_26 | 52.24285 | 0.002 | Appendicularia | *Oikopleura longicauda* | 100 | 100 |
| OTU_819 | 52.30659 | 0.002 | Copepoda | *Clausocalanus furcatus* | 98 | 100 |
| OTU_16 | 51.13282 | 0.0022 | Fungi | *Cladosporium* sp. | 100 | 100 |
| OTU_30 | 49.98707 | 0.0022 | Radiolaria | *Lychnaspis giltschi* | 100 | 100 |
| OTU_23 | 51.3199 | 0.0022 | Copepoda | *Calocalanus* sp. | 100 | 100 |
| OTU_109 | 48.64578 | 0.0022 | Dinoflagellata | *Pelagodinium bei* | 100 | 100 |
| OTU_464 | 49.72527 | 0.0022 | Appendicularia | *Oikopleura longicauda* | 99 | 100 |
| OTU_136 | 50.75547 | 0.0022 | Dinoflagellata | Uncultured Gyrodinium | 100 | 100 |
| OTU_3 | 47.16753 | 0.0033 | Copepoda | *Clausocalanus furcatus* | 100 | 100 |
| OTU_61 | 45.97297 | 0.0057 | Dinoflagellata | Uncultured Duboscquella | 100 | 100 |
| OTU_148 | 45.75592 | 0.0061 | Radiolaria | *Phractopelta dorataspis* | 100 | 100 |
| OTU_39 | 44.93862 | 0.0083 | Radiolaria | *Amphilonche elongata* | 100 | 100 |

|  |  |  |  |  |  |
| --- | --- | --- | --- | --- | --- |

Figure S1. Length distribution of the *Anguilla anguilla* leptocephali investigated.


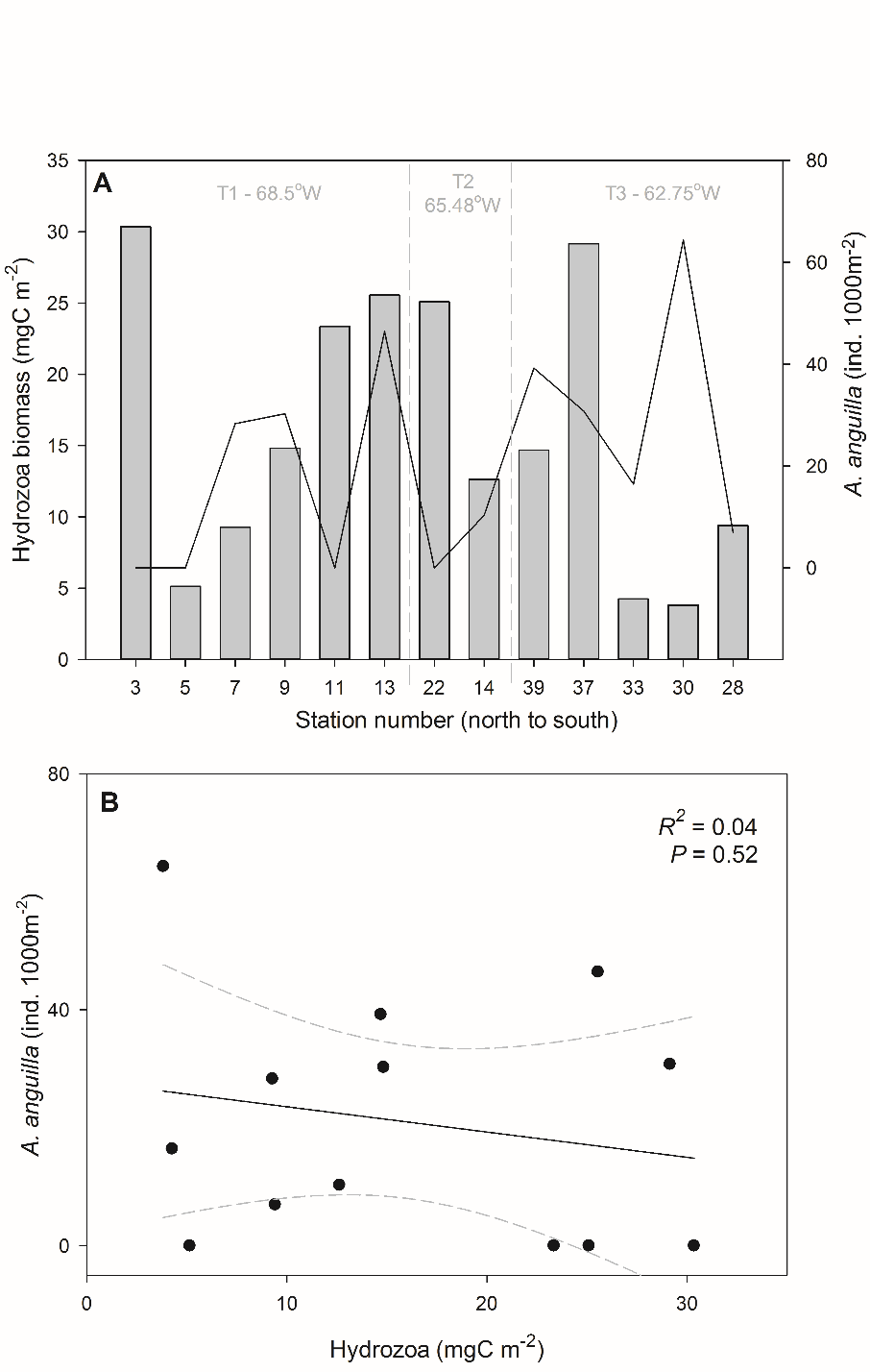


Figure S2. Hydrozoa biomass and European eel larvae abundances in the Sargasso Sea during April 2014. A) Hydrozoa biomass expressed as summed hydromedusae and siphonophore biomass (mgC m^-2^, bars) and *Anguilla anguilla* abundance (ind. 1000 m^-2^, solid line) per station (see figure 1 for reference) along the three transects (T1 – T3). B) Hydrozoa biomass (mgC m^-2^) versus *A. Anguilla* abundance (ind. 1000 m^-2^) showing no significant relationship (linear regression model: R^2^ = 0.04, p=0.52, *n* = 13).


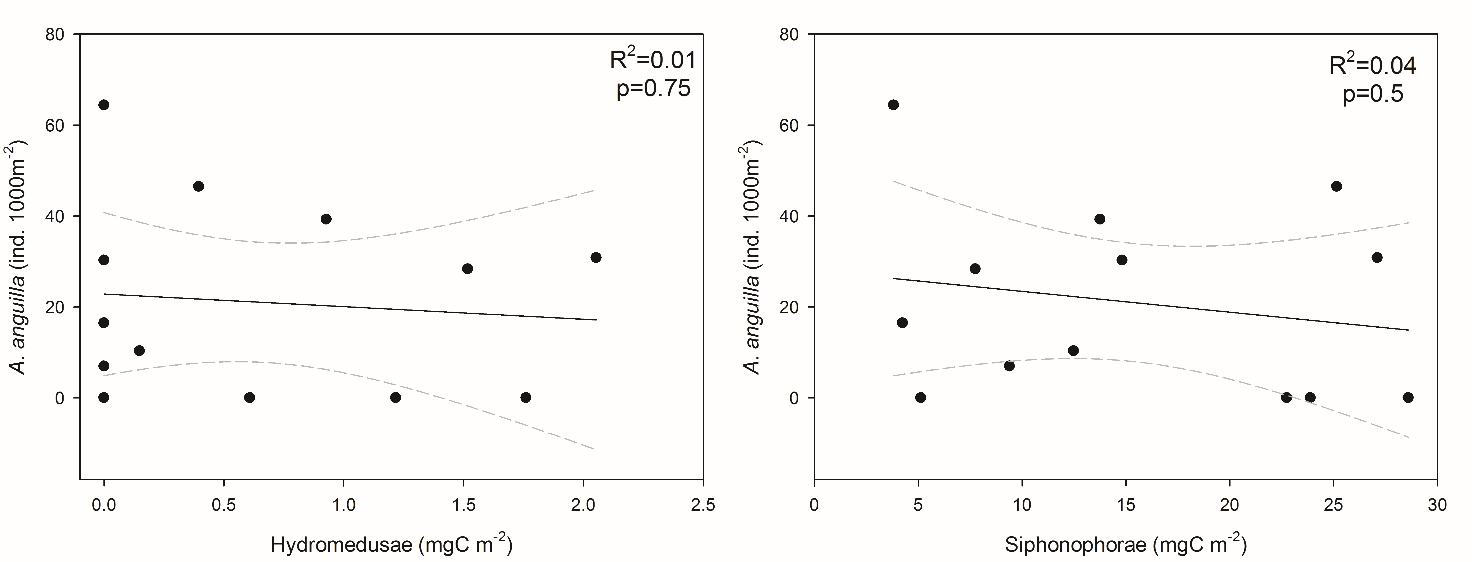


Figure S3. Hydrozoa biomass (mgC m^-2^) differentiated into their major biomass contributors, siphonophorae (right panel) and hydromedusae (left panel) versus *Anguilla anguilla* larvae abundances (1000 m^-2^). Hydromedusae and siphonophorae data originate from 335 µm multinet samples and are integrated for the upper 200 m of the water column, where corresponding eel larvae were caught. *A. anguilla* data originate from MIK net samples. Linear regression (solid line) with 95% confidence bands (dashed line) is indicated for hydromedusae (R^2^ = 0.01, p = 0.75, *n* = 13) and siphonophorae (R^2^ = 0.04, p = 0.5, *n* = 13) showing no significant correlations.


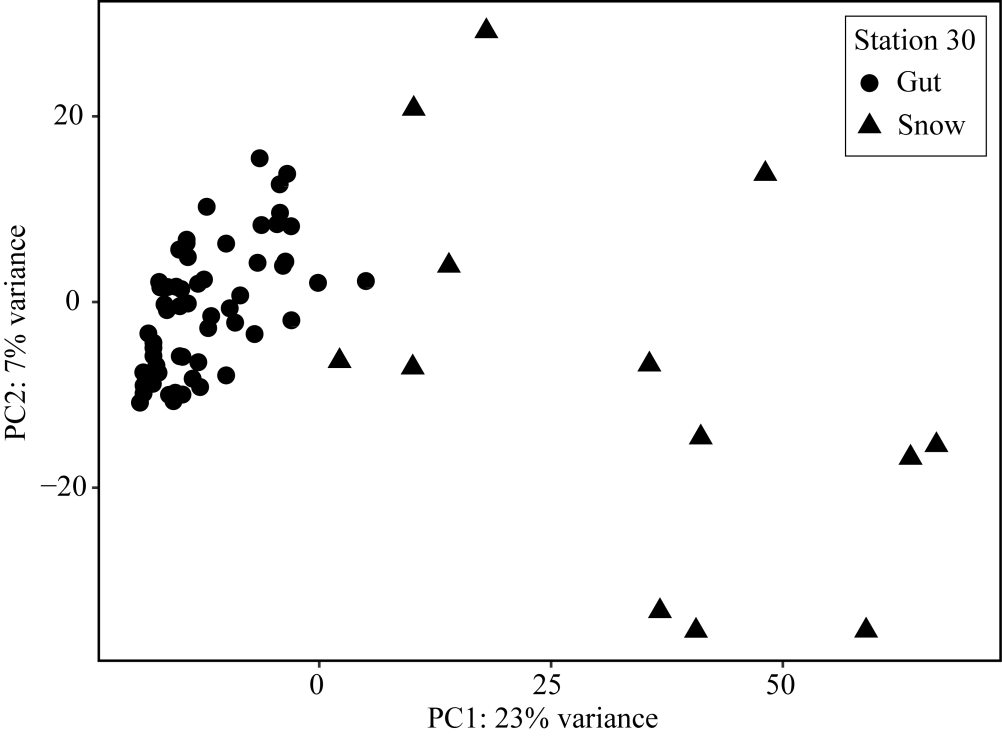


Figure S4. Principal component analyses of *A. anguilla* gut contents and marine snow particle as analyzed by 18S rRNA gene sequencing. Triangles indicate individual leptocephali gut contents and circles indicate individual marine snow aggregates. Plot is like in figure 4 but only with samples from Station 30 shown.


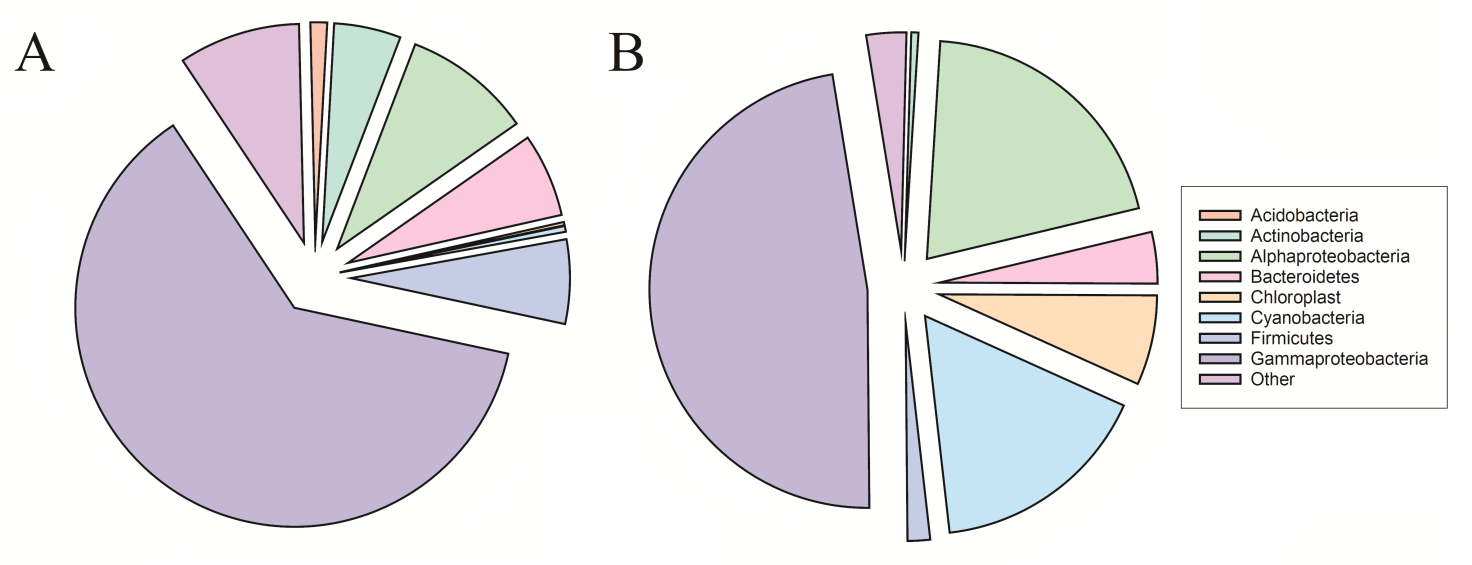


Figure S5. The relative composition of 16S rRNA genes in eel larvae guts (A) and marine snow particles based on Illumina sequencing of extracted DNA. Dominant phyla and sub-phyla are shown.
